# P68 A qualitative evaluation of general practitioners’ views on the use of a Treat Your Respiratory Infection leaflet in general practice in Ireland

**DOI:** 10.1093/jacamr/dlaf230.075

**Published:** 2025-12-04

## Abstract

**Background:**

Respiratory infections (RTIs) account for a high proportion of antibiotic prescribing in general practice, and overprescribing antibiotics is well documented.^1^. The use of patient leaflets during general practitioners’ (GPs) consultations for self-limiting RTIs are promising tools to empower patients to self-manage infections and potentially reduce unnecessary antibiotic prescribing.^2^. A feasibility study was conducted in Ireland to assess the use of a Treat your RTI leaflet in general practice from January to May 2024 (adapted with permission from the UK Health Security Agency TARGET toolkit leaflet). The aim of this study was to understand GPs’ views on using the leaflet during RTI consultations and to inform widescale implementation in general practice.

**Methods:**

Semi-structured interviews were conducted with the nine GPs that participated in the feasibility study of the use of the Treat your RTI leaflet [six based in daytime practice and three in out-of-hours (OOH) settings]. GPs took part in the interview after using the leaflet in 11 to 31 RTI consultations each, primarily when prescribing no antibiotics or delayed antibiotics. The interviews were conducted in-person or online between March and May 2024. Interviews were analysed using inductive thematic analysis, followed by deductive analysis using the Theoretical Domains Framework (TDF) and the Capability, Opportunity, Motivation and Behaviour (COM-B) model for behaviour change (3). Ethical approval was obtained.

**Results:**

GP views mapped to the COM-B model, following thematic analysis and TDF mapping, are as follows. *Physical capability*: Using the leaflet created a structure for the consultation and provided an opportunity to explain delayed antibiotic prescribing. *Psychological capability*: The leaflet supported patient education and GP-patient communication. *Physical opportunity*: All GPs stated the leaflet was clear and easy to read, providing a tangible resource to use in the consultation. The leaflet was perceived as beneficial in GP daytime practice and OOH services, it could be widely used for RTI presentations, for all ages, particularly with parents of young children. One GP stated that busy OOH sessions made using the leaflet more challenging. *Social opportunity*: Recommendations for promoting the leaflet use included using peer champions to promote it through social media and through GP webinars and Continuing Medical Education workshops. *Reflective motivation*: GPs reported that using the leaflet did not increase consultation time. The expected duration of symptoms and safety netting sections were particularly valued. Some GPs reported it influenced their antibiotic prescribing decisions, and it could help to reduce re-consultations for similar presentations. *Automatic motivation*: GPs reported that leaflet use during the consultation provides reassurance that patients are empowered to self-manage RTIs and reduces their antibiotic-seeking behaviour.

**Conclusions:**

The value of using the leaflet to support GP consultations for self-limiting RTIs was clear in both daytime and OOH GP services. Applying the COM-B model for behaviour change identified several facilitators and some barriers to consider when planning wider implementation of use of the leaflet.
